# Supplementary material for: Structure of a eukaryotic cholinephosphotransferase-1 reveals mechanisms of substrate recognition and catalysis
Source: Nat Commun. 2023 May 13;14:2753. doi: 10.1038/s41467-023-38003-9 (PMC10182977; doi:10.1038/s41467-023-38003-9)
Supplement: Supplementary file 1 — Supplementary information [file 41467_2023_38003_MOESM1_ESM.pdf]

Supplementary Figures

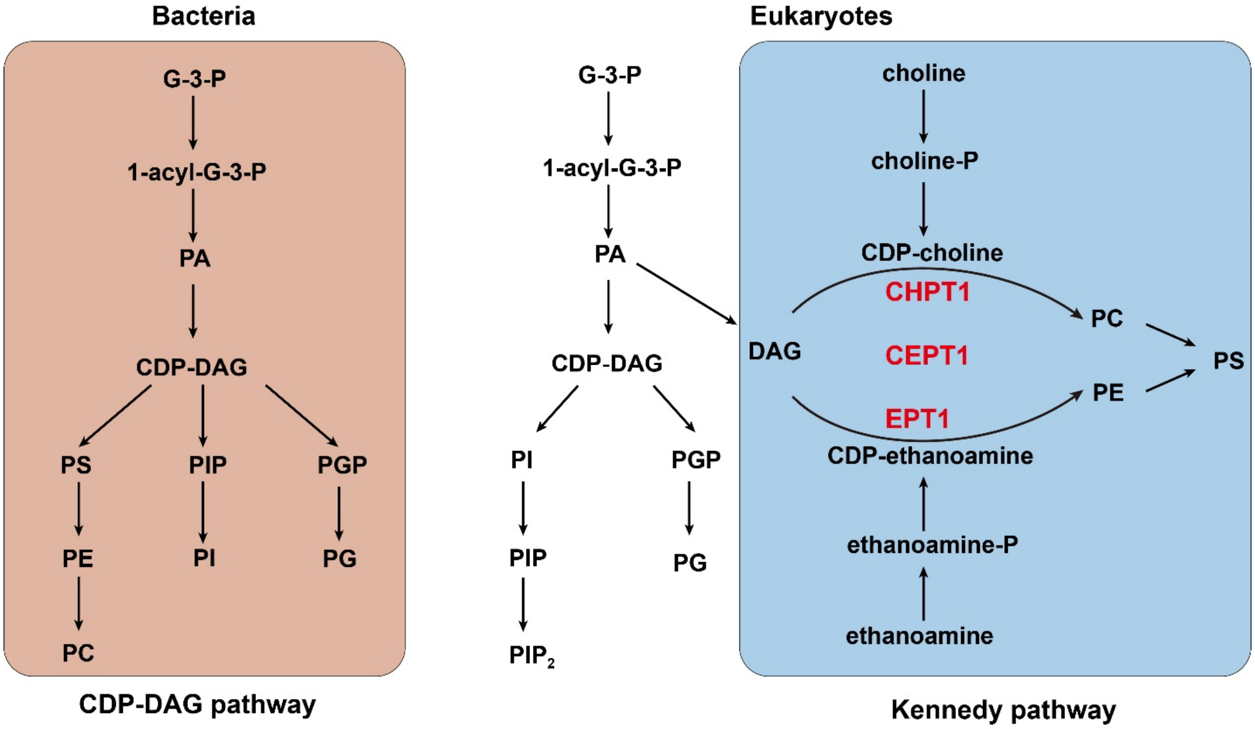

Supplementary Figure 1. Phospholipids synthesis pathways in bacteria and eukaryotic cells. Left: In bacteria, the main pathway for phospholipids synthesis is the CDP-DAG pathway. **Right:** In most of eukaryotic cells, PC, PE and PS are synthesized through the Kennedy pathway. PI and PG are synthesized through the CDP-DAG pathway.

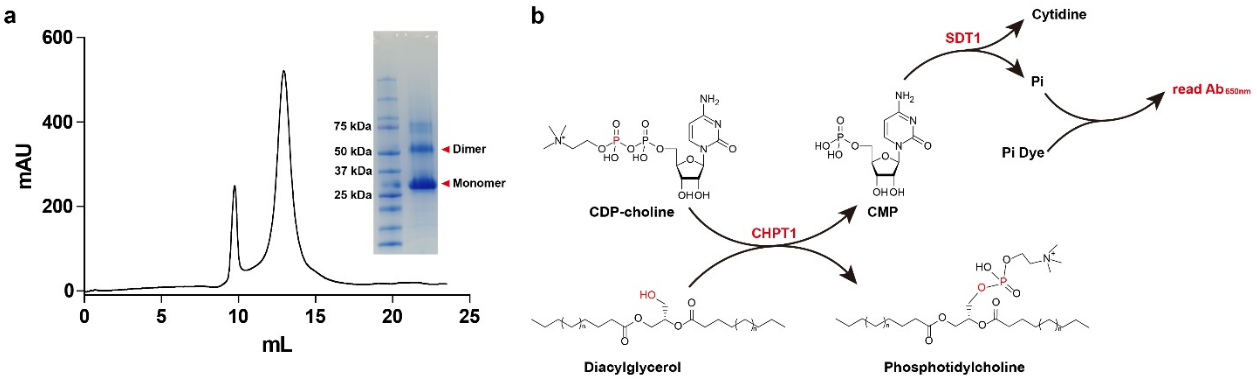

Supplementary Figure 2. Purification and functional assay of xlCHPT1. a. Size-exclusion chromatography profile and SDS-PAGE of xlCHPT1. Both dimer and monomer bands are visible on the SDS-PAGE. The results are representative of over 3 independent repeats of the xlCHPT1 purification experiment. **b.** xlCHPT1 reaction is coupled to that of SDT1 to monitor the production of CMP, which is quantified with the absorption at 650 nm.

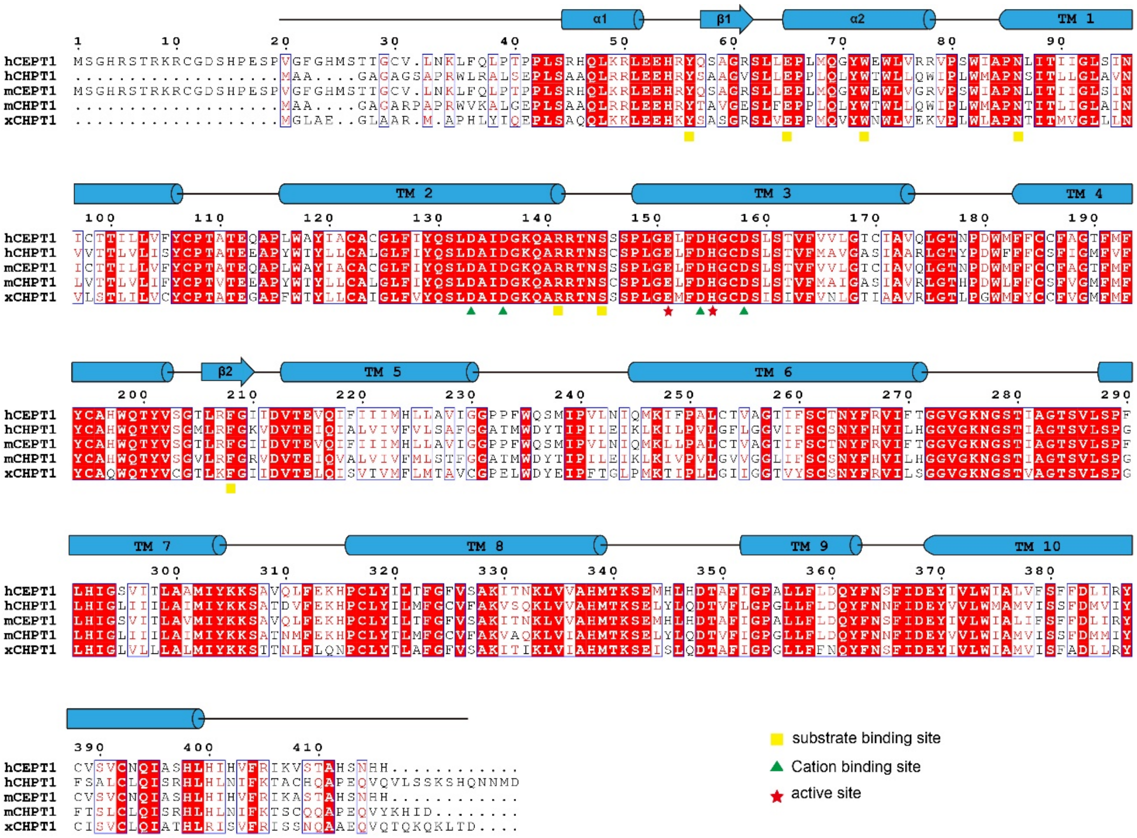

Supplementary Figure 3. Sequence alignment. Human CEPT1(hCEPT1, Q9Y6K0 [https://www.uniprot.org/uniprotkb/Q9Y6K0/entry]), human CHPT1(hCHPT1, Q8WUD6 [https://www.uniprot.org/uniprotkb/Q8WUD6/entry]), mouse CEPT1 (mCEPT1, Q8BGS7 [https://www.uniprot.org/uniprotkb/Q8BGS7/entry]), mouse CHPT1 (mCHPT1, Q8C025 [https://www.uniprot.org/uniprotkb/Q8C025/entry]), frog CHPT1 (xCHPT1, Q4KLV1 [https://www.uniprot.org/uniprotkb/Q4KLV1/entry]) are aligned using the Clustal Omega server[42]. Secondary structural elements of xlCHPT11 are marked above the alignment. Residues are colored based on their conservation using the ESPript server[43]. Residues at the CDP-choline binding site are marked with yellow squares. Cation binding site residues are marked in green triangles. Residues in the active site are marked with red stars.

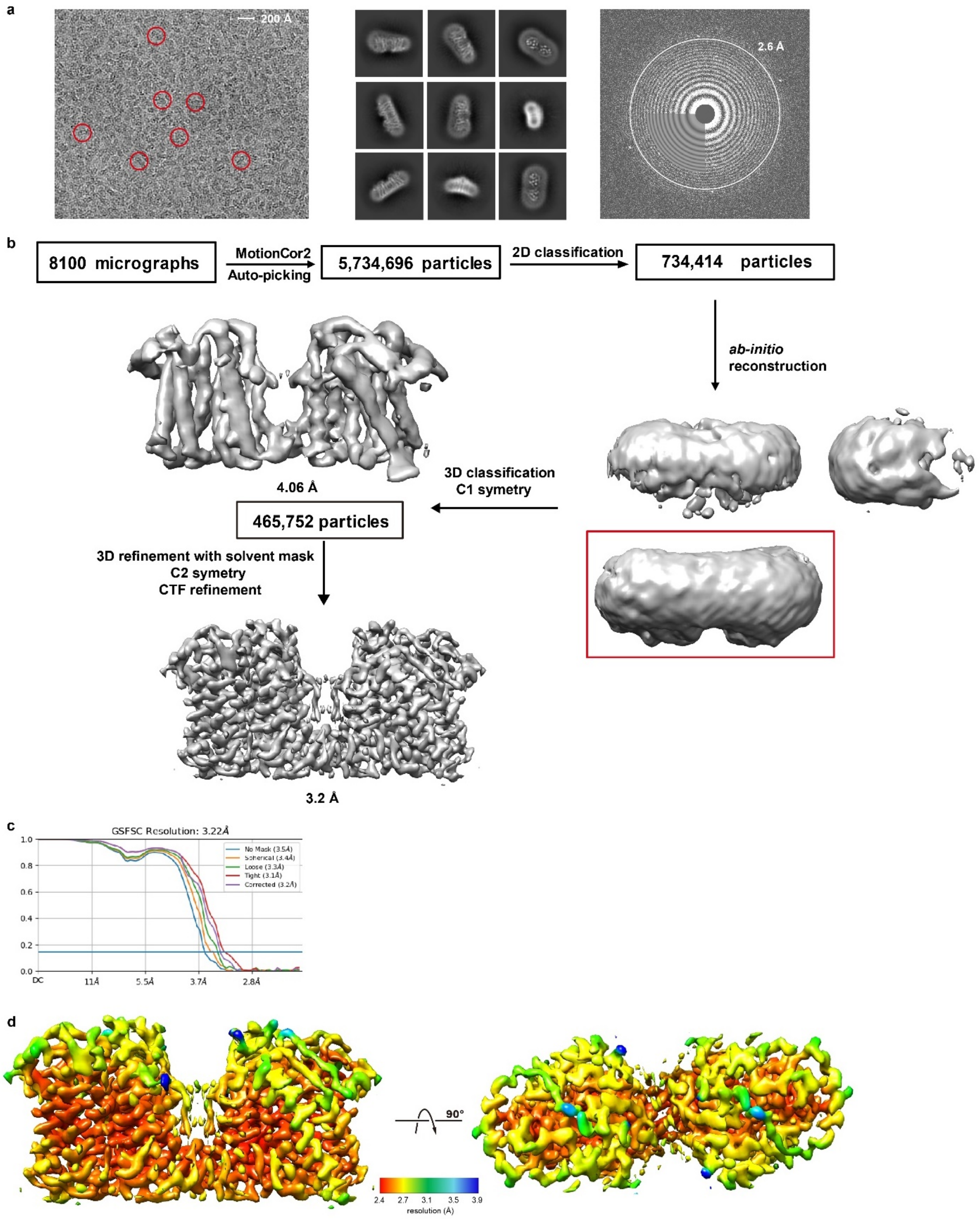

Supplementary Figure 4. Data processing and map resolution of xlCHPT1-Mg^{2+}-CDP data set. a. A representative micrograph of xlCHPT1 (left), its Fourier transform (right) and representative 2D class averages (middle). Representative particles are highlighted in red circles. **b.** A flowchart for data processing and the final map of xlCHPT1. **c.** The gold-standard Fourier shell correlation curve for the final map. **d.** Local resolution map of xlCHPT1 shown in two orientations.

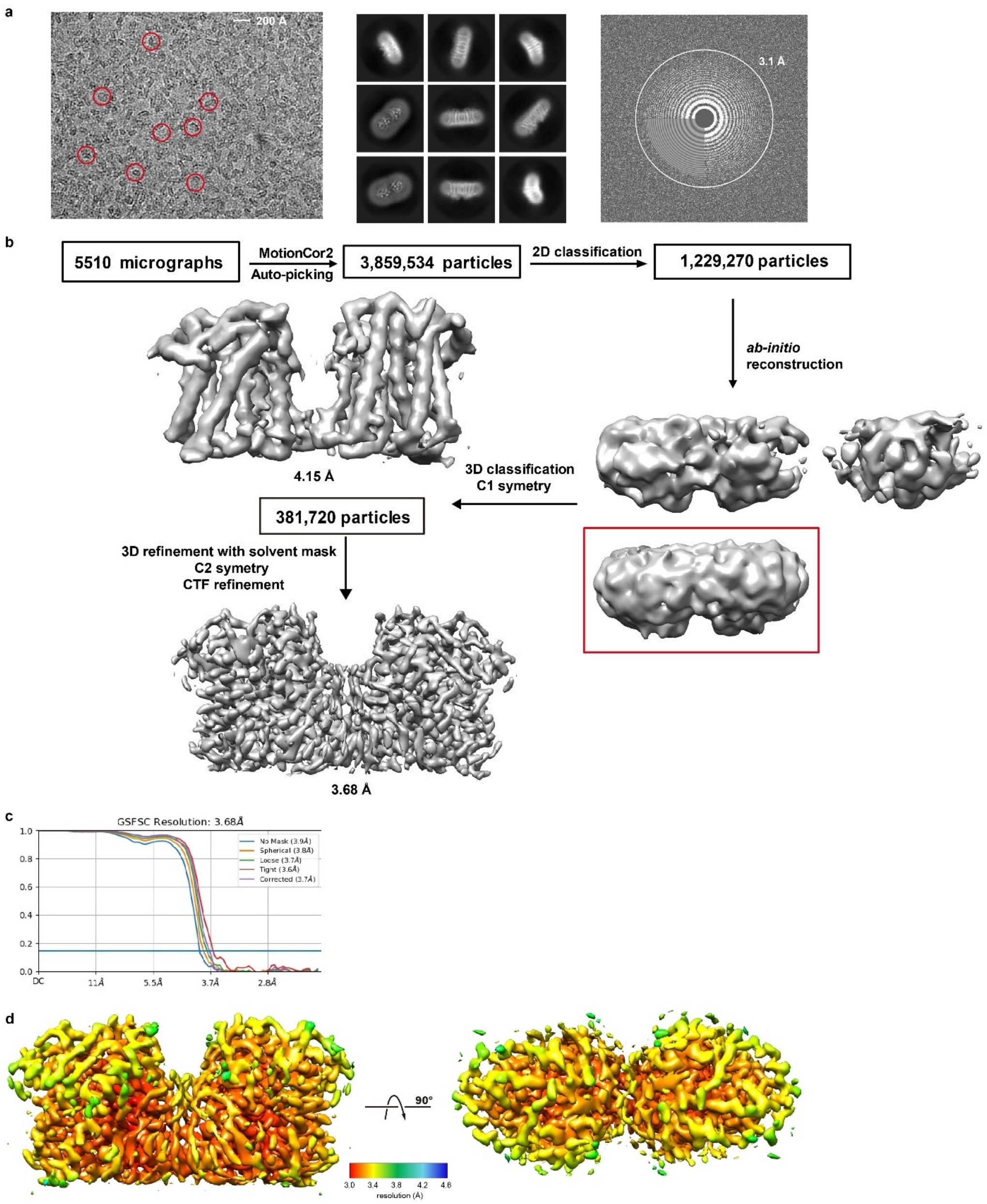

Supplementary Figure 5. Data processing and map resolution of xlCHPT1-Mg^{2+}-CDP-choline data set. a. A representative micrograph of xlCHPT1 (left), its Fourier transform (right) and representative 2D class averages (middle). Representative particles are highlighted in red circles. **b.** A flowchart for data processing and the final map of xlCHPT1. **c.** The gold-standard Fourier shell correlation curve for the final map. **d.** Local resolution map of xlCHPT1 shown in two orientations.

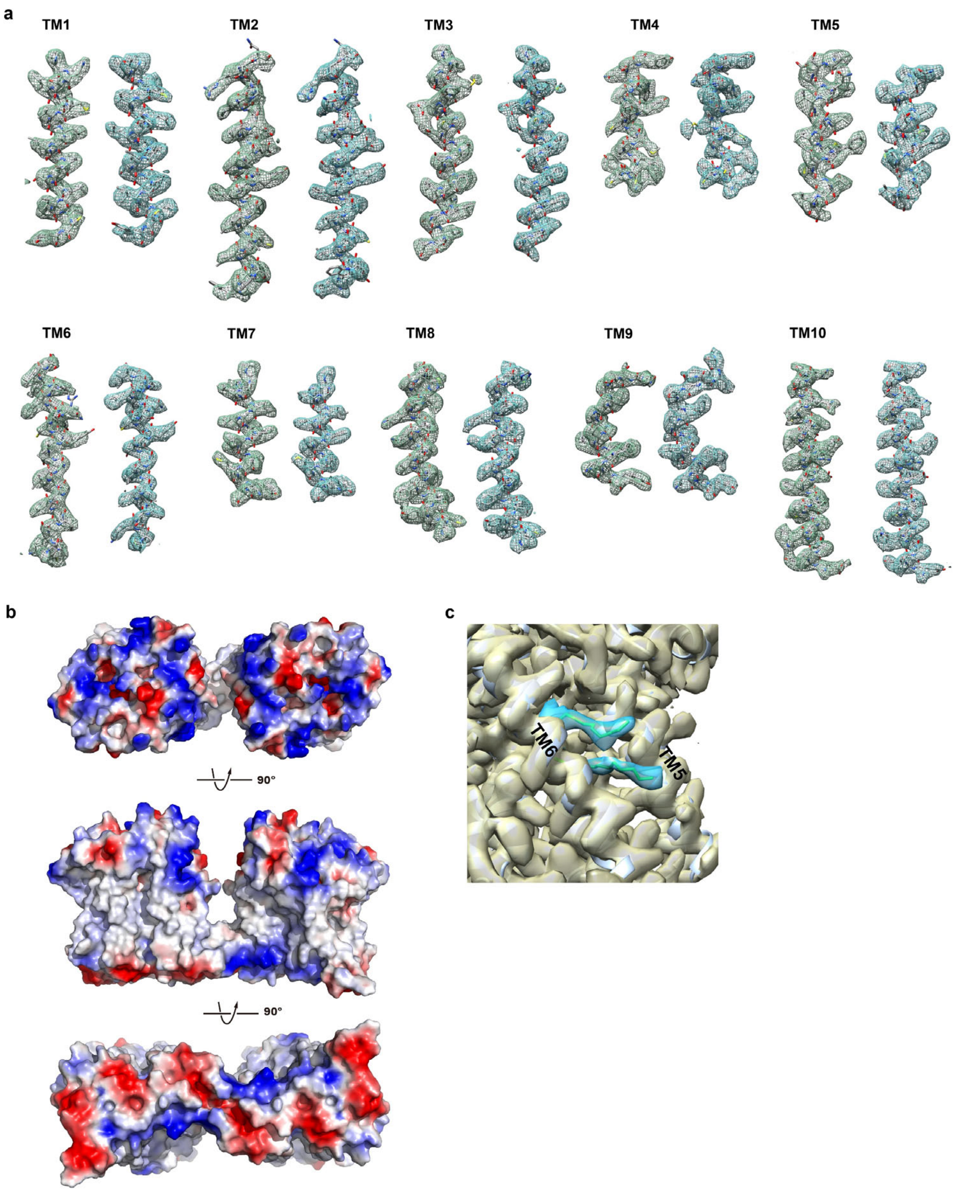

Supplementary Figure 6. Density maps and structural model of xlCHPT1. a. Individual secondary structures of xlCHPT1 are shown as gray sticks, contoured in their density. Density of the xlCHPT1-Mg^{2+}-CDP data set is shown green mesh and that of xlCHPT1-Mg^{2+}-CDP-choline data set is shown in blue mesh. **b.** Electrostatic surface representations of xlCHPT1 dimer in three orientations. The electrostatic potential is calculated using the APBS plugin from Pymol[44]. **c.** A non-protein density (blue surface) in the slit between TM5 and TM6 sufficient for two acyl chains. The xlCHPT1 protein density is shown as yellow surface.

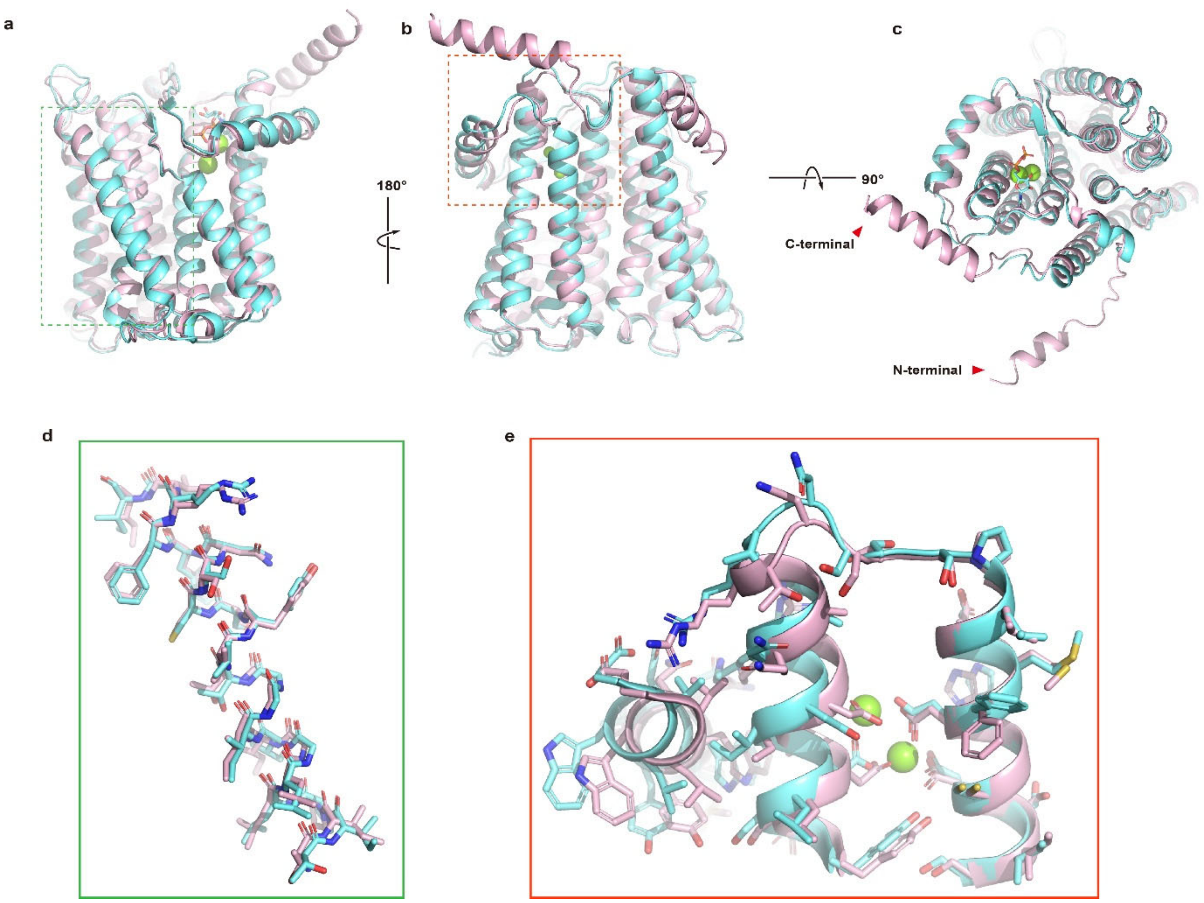

Supplementary Figure 7. Alignment of xlCHPT1 structure and alphafold predicted model. a-c. Overall comparison of xlCHPT1 structure (cyan) with alphafold predicted model (pink) in three orientations. **d-e**. Detailed residues alignment of the highlighted helix in panel **a** and panel **b**. Residues are shown in sticks.

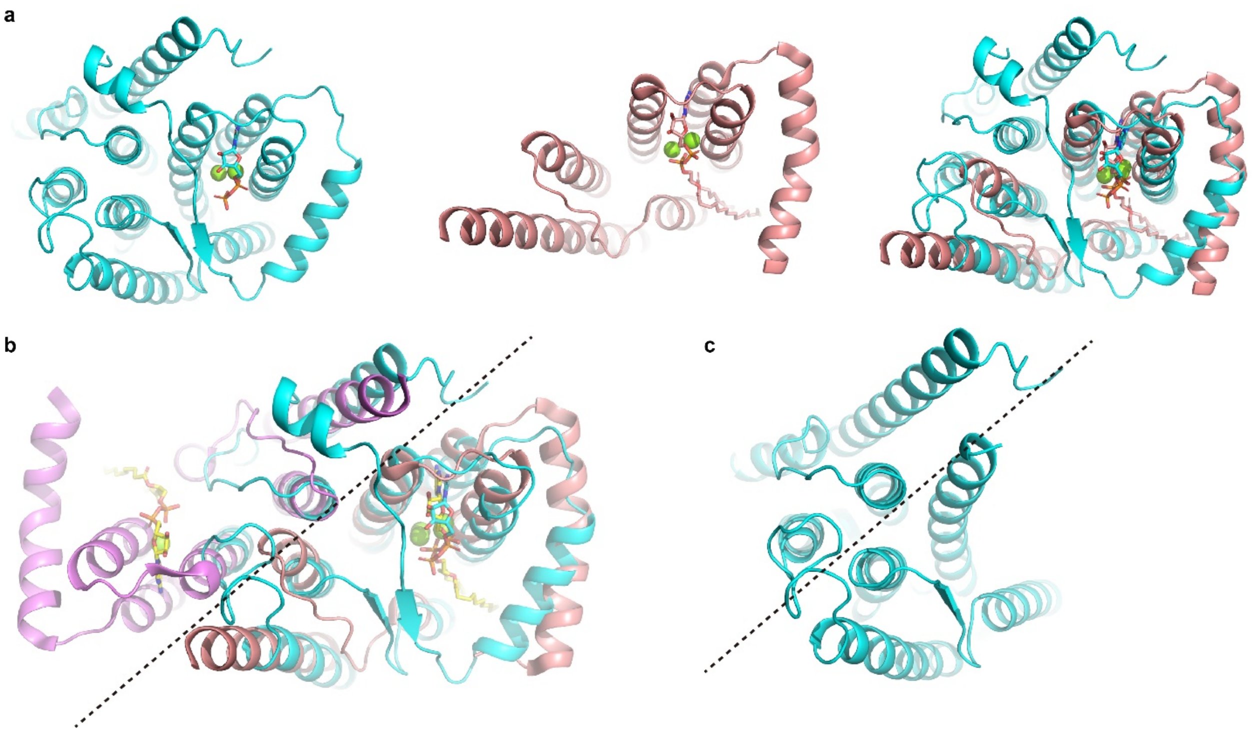

Supplementary Figure 8. Structural symmetry in xlCHPT1. a. Structure alignment of xlCHPT1 (cyan) and RsPIPS (5D92 [http://doi.org/10.2210/pdb5D92/pdb], pink). **b.** Structural alignment of xlCHPT1 monomer (cyan) with RsPIPS dimer (pink and purple). **c.** Structural symmetry between TM3-6 and TM7-10 of xlCHPT1.

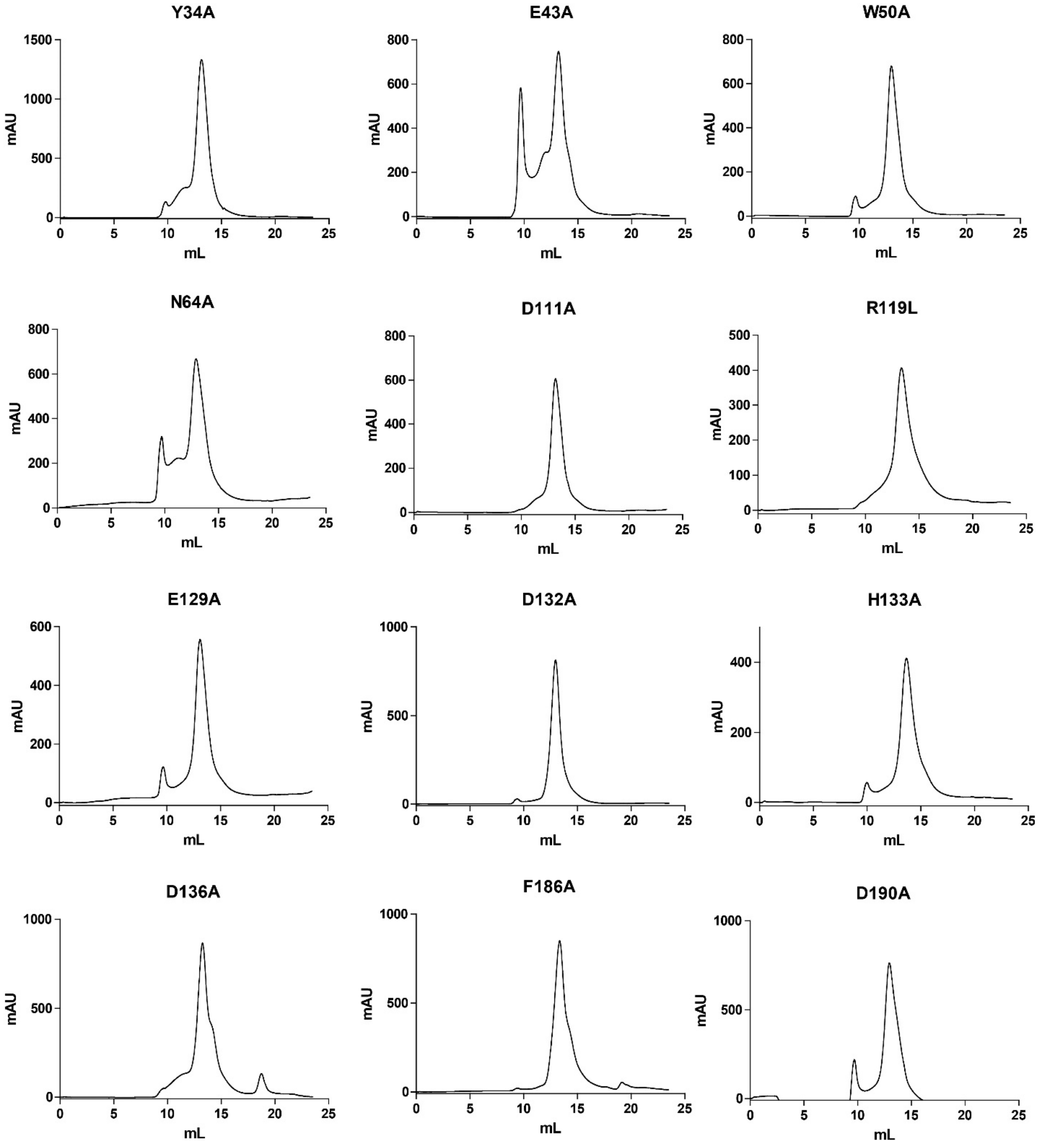

Supplementary Figure 9. Size-exclusion chromatography profiles of xlCHPT1 mutants.

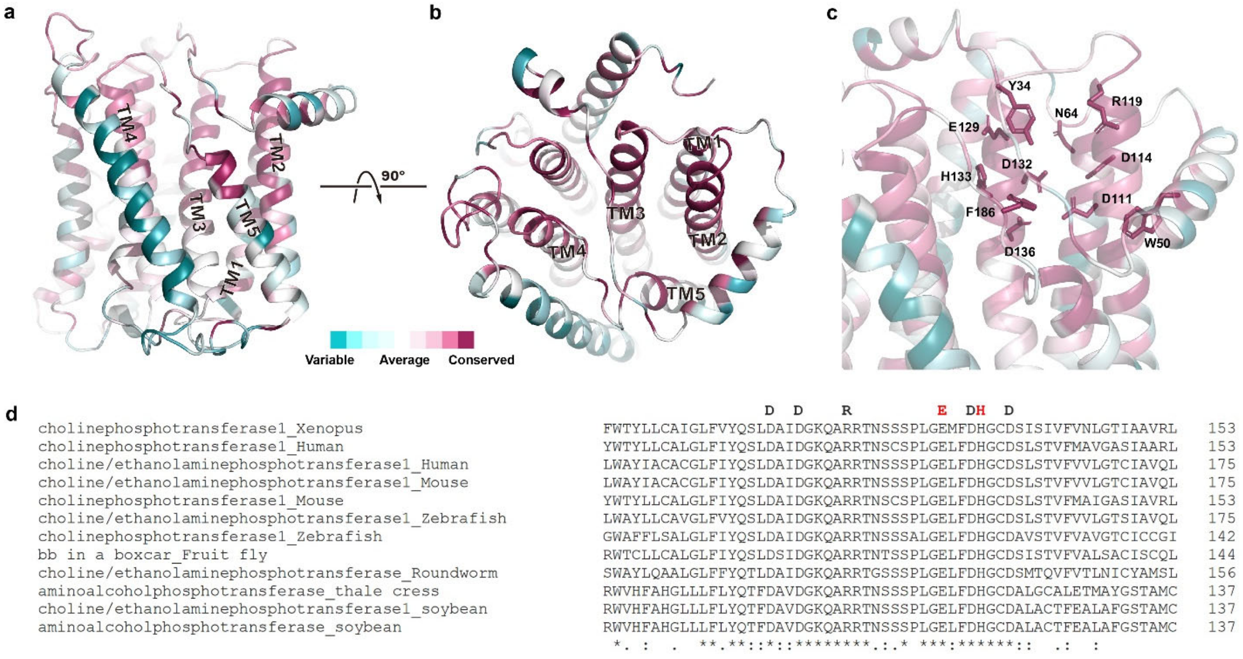

Supplementary Figure 10. Conservation of substrate binding site and catalytic site in eukaryotic CDP-APs. **a.** xlCHPT1 monomer is colored on the basis of the conservation score of each residue, calculated by the ConSurf server[45]. Residues in substrate binding sites are shown as sticks. **b.** Signature motif alignment of CDP-APs from human (NP_001007795.1 [https://www.ncbi.nlm.nih.gov/protein/NP_001007795] , NP_064629.2 [https://www.ncbi.nlm.nih.gov/protein/NP_064629.2]), mouse (NP_001344747.1 [https://www.ncbi.nlm.nih.gov/protein/NP_001344747.1], NP_001140162.1 [https://www.ncbi.nlm.nih.gov/protein/NP_001140162.1]), xenopus (NP_001089575.1 [https://www.ncbi.nlm.nih.gov/protein/NP_001089575.1]), zebrafish (XP_021325046.1 [https://www.ncbi.nlm.nih.gov/protein/XP_021325046.1], NP_997789.1 [https://www.ncbi.nlm.nih.gov/protein/NP_997789.1]), roundworm (NP_001257200.1 [https://www.ncbi.nlm.nih.gov/protein/NP_001257200.1]), fruit fly (NP_725301.1 [https://www.ncbi.nlm.nih.gov/protein/NP_725301.1]), thale cress (NP_001030767.1 [https://www.ncbi.nlm.nih.gov/protein/NP_001030767.1]) and soy bean (XP_003518818.1 [https://www.ncbi.nlm.nih.gov/protein/XP_003518818.1], NP_001238284.2

[https://www.ncbi.nlm.nih.gov/protein/NP_001238284.2]). The conserved CDP-AP signature motif and proposed catalytic site residues are highlighted.

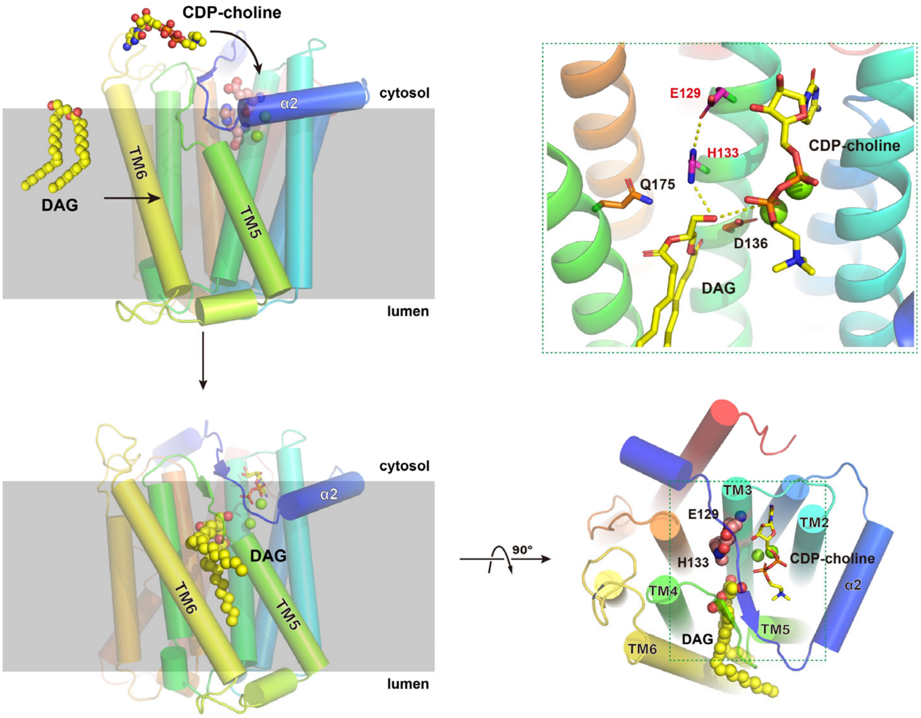

Supplementary Figure 11. Proposed entry and docking of DAG in xlCHPT1. xlCHPT1 is shown as cartoon, DAG and CDP-choline are shown as yellow spheres or sticks. Active site residues, E129 and H133, are shown as sticks or spheres. Residues that may interact with DAG are shown as orange sticks.

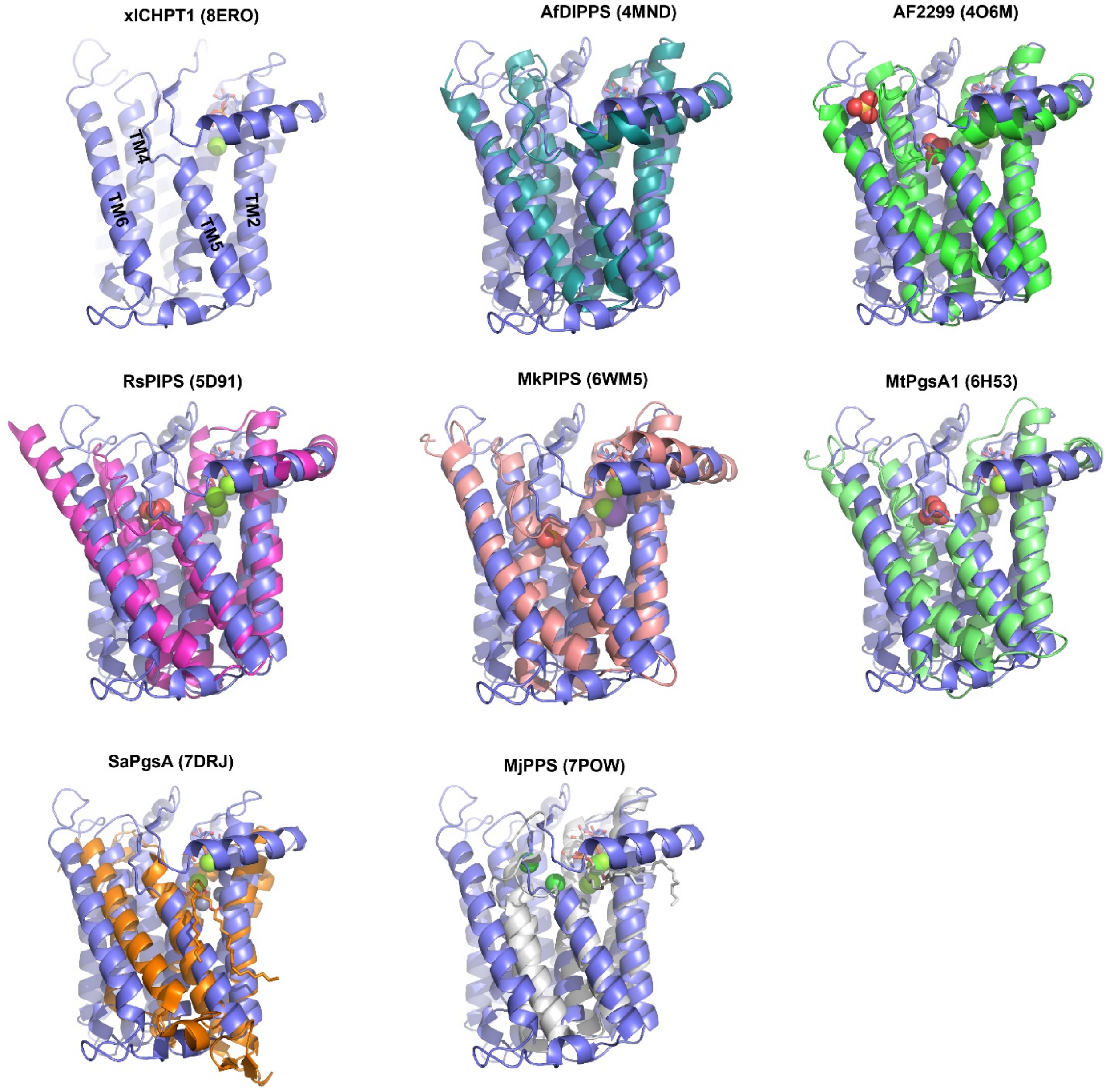

Supplementary Figure 12. Structural comparison of xlCHPT1 and bacteria CDP-APs. Structures of xlCHPT1 is shown as purple cartoon. All other bacteria CDP-APs are shown in other different colors.

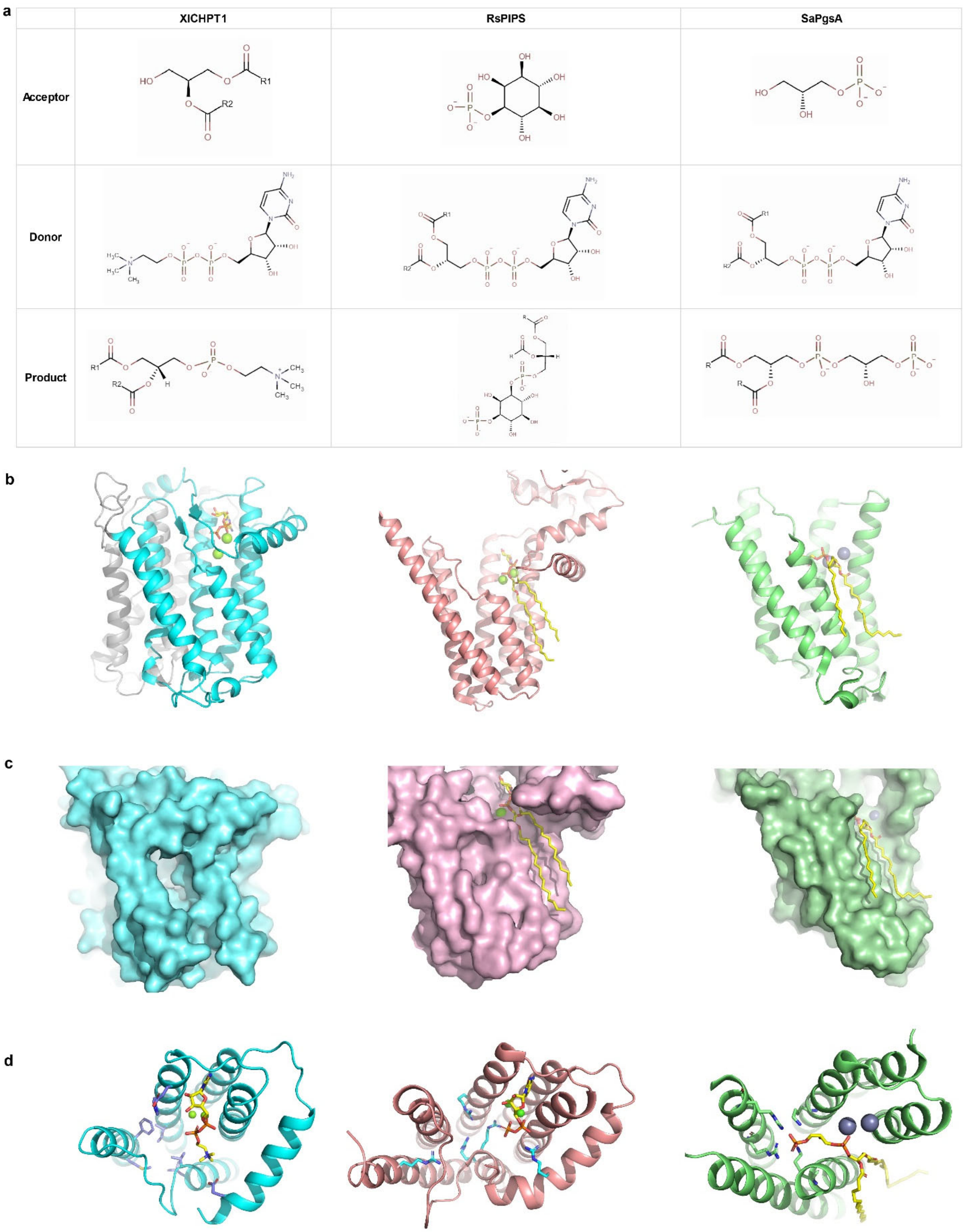
a
XlCHPT1
RsPIPS
SaPgsA
Acceptor
Donor
Product
b
c
d

Supplementary Figure 13. Structures comparison of xlCHPT1, bacterial RsPIPS (pdb code 5D92 [http://doi.org/10.2210/pdb5D92/pdb]) and SaPgsA (pdb code 7DRJ [http://doi.org/10.2210/pdb7DRJ/pdb]). a. Substrates and products of xlCHPT1, RsPIPS and SaPgsA**. b.** Structure of xlCHPT1 that highlights CDP-choline, structure of RsPIPS that highlights CDP-DAG and structure of SaPgsA that highlights PGP. **c.** Space-filled representation of xlCHPT1, RsPIPS and SaPgsA. xlCHPT1 is shown in cyan, RsPIPS is in pink, and SaPgsA is in green. **d.** Comparison of DAG or inositol phosphate binding sites in the three structures, with relevant residues shown as sticks.

Supplementary Table 1. Summary of cryo-EM data collection, processing, and structural refinement

	xlCHPT1-Mg^{2+}- CDP-choline (EMD-28557) (PDB 8ERP)	xlCHPT1-Mg^{2+}- CDP (EMD- 28556) (PDB 8ERO)
**Data collection and processing**		
Magnification	81,000	81,000
Voltage (kV)	300	300
Electron exposure (e–/Å^2)	50	50
Defocus range (μm)	[-2.0, -0.8]	[-2.0, -0.8]
Pixel size (Å)	1.1	1.1
Symmetry imposed	C2	C2
Initial particle images (no.)	3,859,534	5,734,696
Final particle images (no.)	381,720	465,752
Map resolution (Å)	3.7	3.2
FSC threshold	0.143	0.143
Map resolution range (Å)	2.9-4.6	2.4-4.0
**Refinement**		
Initial model used (PDB code)	8ERP	8ERO
Model resolution (Å)	3.8	3.4
FSC threshold	0.5	0.5
Model resolution range (Å)	3.5-3.9	2.8-3.5
Map sharpening *B* factor (Å^2)	-100	-100
Model composition		
Non-hydrogen atoms	6058	6156
Protein residues	728	728
Ligands	26	32
B factors (Å^2)		
Protein	35.5	34.6
Ligand	24.8	44.5
R.m.s. deviations		
Bond lengths (Å)	0.003	0.003
Bond angles (°)	0.729	0.639
Validation		
MolProbity score	1.79	1.80
Clashscore	7.21	5.36
Poor rotamers (%)	0	0
Ramachandran plot		
Favored (%)	94.2	91.2
Allowed (%)	5.8	8.8
Disallowed (%)	0	0

Supplementary Table 2. Sequence of primers

Oligo name	Oligo sequence (5' to 3')
Y34A-F	GAGGAACACAAGGCTTCCGCTTCT
Y34A-R	CTTGTGTTCCTCCAGCTTCTTCAG
E43L-F	AGGTCTCTCGTTCTCCCCCCTATG
E43L-R	AACGAGAGACCTTCCAGAAGCGGA
W50A-F	ATGCAGGTGTACGCTAACTGGTTG
W50A-R	GTACACCTGCATAGGGGGCTCAAC
N64V-F	TGGCTGGCCCCGGTCACAATCACC
N64V-R	CGGGGCCAGCCACAATGGAACCTT
D111A-F	TACCAGAGCCTCGCTGCTATCGAT
D111A-R	GAGGCTCTGGTACACGAACAGGCC
R119L-F	GGAAAGCAAGCCCTCCGCACCAAC
R119L-R	GGCTTGCTTTCCATCGATAGCGTC
E129A-F	AGCCCGTTGGGAGCTATGTTCGAC
E129A-R	TCCCAACGGGCTAGAGGAGTTGGT
D132A-F	GGAGAAATGTTCGCTCACGGTTGC
D132A-R	GAACATTTCTCCCAACGGGCTAGA
H133A-F	GAAATGTTCGACGCTGGTTGCGAT
H133A-R	GTCGAACATTTCTCCCAACGGGCT
D136A-F	GACCACGGTTGCGCTAGCATCTCA
D136A-R	GCAACCGTGGTCGAACATTTCTCC
F186A-F	GGTACCCTGAAGGCTGGCATCATC
F186A-R	CTTCAGGGTACCACACACGTATGT

References:

42. Dineen, S. A. *et al.* Fast, scalable generation of high-quality protein multiple sequence alignments using Clustal Omega. *Mol. Syst. Biol.* **7,** 539 (2011).

43. Robert, X. & Gouet, P. Deciphering key features in protein structures with the new ENDscript server. *Nucleic Acids Res.* **42,** (2014).

44. Baker, N. A., Sept, D., Joseph, S., Holst, M. J. & McCammon, J. A. Electrostatics of nanosystems: Application to microtubules and the ribosome. *Proc. Natl. Acad. Sci. U. S. A.* **98,** 10037–10041 (2001).

45. Landau, M. *et al.* ConSurf 2005: The projection of evolutionary conservation scores of residues on protein structures. *Nucleic Acids Res.* **33,** (2005).
